# The mechanism of digital feedback on health information anxiety among older adults: information processing self-efficacy as a mediating variable

**DOI:** 10.3389/fpubh.2025.1676970

**Published:** 2025-11-05

**Authors:** Yang Zhu, Xin Wang, Xiao Zhang, Yan Li, Yepeng Chen

**Affiliations:** ^1^School of Economics and Management, Beijing University of Posts and Telecommunications, Beijing, China; ^2^China Internet Network Information Center, Beijing, China; ^3^Chaoyang District Human Resources Public Service Center of Beijing Municipality, Beijing, China; ^4^School of Economics and Management, Anhui University of Science and Technology, Huainan, Anhui, China

**Keywords:** digital feedback, information processing self-efficacy, health information anxiety, older adult digital divide, mediating mechanism

## Abstract

**Background:**

Digital feedback emerges as a significant variable influencing health information anxiety among older adults, and information processing self-efficacy also plays a crucial role in this process. This study aims to clarify the logical relationships among digital feedback, health information anxiety, and information processing self-efficacy.

**Methods:**

Guided by the “hypothesis testing” paradigm, this empirical study was based on the construction of a mediation model to examine how digital feedback influences health information anxiety among older adults. Stratified random sampling in conjunction with probability proportional to size (PPS) sampling was used to survey a sample of 1,713 older adults from 30 Chinese cities. Exploratory factor analysis (EFA), correlation analysis, causal steps approach, and the Bootstrap method were employed to test the mediating model. Mediation analysis was conducted using the PROCESS macro, and in-depth interviews were carried out to explore the underlying mechanisms of this process.

**Results:**

The study found that digital feedback had a negative effect on health information anxiety among older adults (β = −0.396, *p* < 0.001), while it had a positive impact on their information processing self-efficacy (β = 0.700, *p* < 0.001). Additionally, information processing self-efficacy had a negative effect on health information anxiety among older adults (β = −0.401, *p* < 0.001). The analysis further revealed that the relationship between digital feedback and health information anxiety was partially mediated by information processing self-efficacy (β = −0.2806, SE = 0.0157, 95% CI = −0.3115, −0.2503).

**Conclusions:**

Digital feedback not only directly mitigates HIA among older adults but can also indirectly reduce health information anxiety by enhancing their information processing self-efficacy. It should be emphasized that inappropriate digital feedback from children, such as insufficiently thorough instruction or lack of patience, may exacerbate health information anxiety among older adults. Therefore, children should actively participate in the digital feedback process, demonstrate patience during feedback, and provide targeted assistance based on the actual needs of older adults. This approach can help older adults maintain their physical and mental wellbeing while better facilitating their integration into the digital society.

## 1 Introduction

In the environment of overlapping population aging and societal digitization, an increasing number of older adults experience anxiety regarding health information due to experiencing digital divide challenges. The world currently faces the challenge of the convergence between population aging and societal digitization. According to publicly available data from the World Health Organization, the global population aged 60 and over had already exceeded 1 billion by 2019 and is projected to reach 1.4 billion by 2030 (1). Simultaneously, internet-based digital products and services have permeated daily life, work, and other social environments. By 2023, global internet users surpassed 5 billion, accounting for over 60% of the world's total population, with particularly pronounced growth observed among older adults (2). With aging and changes in health status, the demand for health information among older adults has significantly increased. However, the existence of the digital divide severely hinders their effective access to and utilization of such information. On one hand, some older adults struggle to obtain or are reluctant to use digital devices, continuing to rely on traditional media for health information (3). On the other hand, due to age-related cognitive decline, even when they do have access to digital technologies, older adults often find it difficult to master them proficiently, facing challenges in searching for, filtering, applying, and sharing health information (4). Moreover, the general lack of age-friendly design in existing applications further increases the difficulty of using digital devices (5). More critically, older adults lag behind in digital thinking. When faced with a large amount of health information whose authenticity is difficult to discern, they are more likely to be misled by rumors or fall victim to online fraud (6). These obstacles not only lead to the gradual marginalization of older adults in the information ecosystem but also easily trigger negative emotions such as anxiety, thereby damaging their mental health (7).

In response to this challenge, many countries and regions worldwide have elevated the improvement of older adults' e-health literacy to a key public policy agenda and are actively exploring intervention strategies. In the United States, a diverse and comprehensive system for cultivating citizens' digital literacy has been established through coordinated efforts among government agencies, educational institutions, and social organizations, with some libraries specifically offering e-health literacy education courses for older adults (8). However, research indicates that significant disparities in the digital divide persist across different racial groups and regions (9). The European Union, similar to the United States, emphasizes legal and policy interventions to collectively address the challenges of the digital divide (10), with particular attention to enhancing older adults' digital competence. To this end, specialized task forces have been established to provide technical and device support to older adults and to design customized training programs (11). Despite continuous improvements in internet access rates among older adults driven by the implementation of the “Digital Europe Programme,” only 44.0% of individuals aged 65 and above report possessing essential digital skills (12). Japan, unlike the United States and the European Union, emphasizes respecting individual differences in capability and advocates enhancing digital literacy through autonomous practice (13), with a noticeable decline in internet usage among older adults as age increases (14). Notably, existing international research primarily focuses on the impact of formal education or policy-driven interventions on older adults' e-health literacy, aiming to bridge the digital divide and promote mental health in later life (15–18), while largely overlooking the significant potential of informal family-based learning—digital feedback—in this process.

As an important approach to bridging the digital divide, digital feedback not only improves digital skills and literacy among older adults (19), but also helps them experience familial warmth, enhances their self-confidence and sense of achievement (20), and thereby reduces anxiety. In China, the acquisition of digital devices and skills among older adults is predominantly facilitated by family members (21, 22). Nevertheless, few studies have explored the impact of digital feedback on health information anxiety in this population. Furthermore, self-efficacy plays a pivotal role in this process. Self-efficacy has been shown in numerous empirical investigations to have a negative correlation with anxiety and to be beneficial to mental and physical health (23). An innovative study found that older adults who took part in computer training at a public library showed significantly lower levels of computer-related anxiety and much higher levels of interest and self-efficacy (24). Currently, no study has incorporated “digital feedback,” “self-efficacy,” and “health information anxiety” into a unified theoretical framework, particularly lacking systematic empirical examination of the pathways among these three factors. Therefore, further investigation into the underlying mechanisms through which digital feedback influences health information anxiety among older adults is of significant importance.

Therefore, this study is designed to answer the following two research questions: (1) How does digital feedback influence health information anxiety in older adults? (2) How does information processing self-efficacy serve as a mediating factor in this process? This research endeavors to clarify the relationship between digital feedback, information processing self-efficacy, and health information anxiety to support older adults in integrating into digital life.

## 2 Literature review and hypotheses

### 2.1 Digital feedback

In 1988, scholar Zhou Xiaohong first proposed the concept of cultural feedback, describing it as the process by which the older generation absorbs cultural elements from the younger generation during periods of rapid cultural change (25). Digital feedback represents the primary manifestation of cultural feedback in the digital age (26). Scholar Zhou Yuqiong noted that over the past three decades, cultural feedback related to digital media has evolved into a new dimension approximately every decade, progressing from material feedback (1990s) to skill-based feedback (2000s) and subsequently to ideational feedback (2010s). She defined digital feedback as “the process by which younger generations mentor older generations in digital access, use, and literacy (19),” a definition that has since become foundational in subsequent research.

Along with conceptual development and improvement, numerous scholars have validated children's role in shaping their parents' use of digital devices. For example, Nelissen et al. established that parents often rely on children's instruction for digital media (27), and Kiesler et al. (28) documented an informal transfer of computer competence from younger to older family members. Also, academics have labeled younger generation “young experts” and “digital natives” (29). Digital feedback helps older adults better integrate into digital environments, yet existing research remains limited, primarily focusing on the influencing factors and outcomes of digital feedback. Some researchers have also looked at more specialized topics, such as older adults' use of shorter video platforms and WeChat. Digital feedback is a crucial approach to bridge the digital divide and improve emotional ties among families, according to research (30). Families with higher socioeconomic status are more likely to offer technological assistance (31). In contrast, older adults who are younger, better educated, and maintain frequent family interactions are more responsive to digital feedback (32). Older adults can become proficient in digital technology, improve everyday convenience, promote intergenerational harmony, and lessen feelings of social isolation and loneliness through this process (7).

### 2.2 Health information anxiety

Two perspectives define health information anxiety: one views Cyberchondria as a form of health information anxiety, pointing out that people may look up health information online when they are worried about their health and that ambiguous or deceptive information frequently makes anxiety worse (33). Li et al. proposed that online health anxiety aligns with the core of Cyberchondria, suggesting they represent opposite ends of a symptom continuum (34). The other regards health information anxiety as a subset of information anxiety. Wurman first proposed information anxiety, describing it as a “black hole” between data and knowledge that emerges when information fails to meet individuals' needs (35). Scholars in China also note that information anxiety can arise at various stages of information acquisition and use, characterizing it as a complex emotional response—including nervousness, worry, fear, panic, and discomfort—triggered by external factors (e.g., information quality, environmental conditions) and internal factors (e.g., information literacy, personality traits) (36).

This study investigates the mechanisms through which digital feedback influences health information anxiety among older adults, adopting the second definition for this purpose. Additionally, research further highlights that older adults are particularly concerned about information related to disease prevention, medical consultation (37), treatment (38), and nutrition (39). Accordingly, health information is classified into four domains: medical services (e.g., disease consultation, telemedicine), rehabilitation (e.g., medication, rehabilitation precautions), disease prevention (e.g., disease etiology, preventive measures, early symptoms), and health preservation (e.g., dietary hygiene, nutrition) to address the health maintenance needs of older adults. Based on this, the present study defines health information anxiety as the psychological state of unease, tension, and worry that arises when individuals, in the information age, seek to maintain their health by accessing and using online health information—including medical services, rehabilitation treatments, disease prevention, and wellness promotion—but are unable to effectively search for, filter, and utilize the vast amount of available health information due to the influence of external factors (such as information environment and information quality) and internal factors (such as self-perception and information literacy).

A substantial body of research has focused on the health information-seeking behaviors of older adults. Compared to other age groups, older adults exhibit heightened attention to health information, yet they face significant digital divide challenges due to deteriorating physical and cognitive functions (24). Research has identified factors such as information alienation, retrieval system quality, and information use environment as contributors to user anxiety (40, 41). Specifically, older adults with advanced age, low education, poor health (34), low uncertainty tolerance, and negative cognitive tendencies (42) are more susceptible to health information anxiety—an emotional state that may further lead to health information avoidance behaviors (43).

### 2.3 Digital feedback and health information anxiety

Social support refers to the material and psychological assistance that individuals receive from their social relationships (44), serving as an external resource upon which individuals can rely when facing stress or adversity (45). Previous studies have applied social support theory to promote health information behaviors, indicating that families play a critical role in providing such support (46). In particular, support from children has demonstrated significant effects in improving internet use among older adults, enhancing their mental health, and reducing their tendency to avoid health-related information (47).

This suggests that, digital feedback, as a form of family-based social support, also plays an important role in alleviating health information anxiety among older adults, with children's feedback showing particularly notable effects (30). First, children improve digital accessibility for older adults by providing devices such as smartphones, thereby creating conditions for full engagement with digital life (48). Second, through daily instruction and systematic guidance, children help older adults master essential digital skills to overcome health information processing difficulties and reduce information anxiety (49). Finally, children promote the value and convenience of digital technologies to older adults while teaching them about cybersecurity and digital ethics. These efforts collectively foster digital thinking and behavioral patterns among older adults, gradually diminishing their unease with new technologies (50) and enabling better adaptation to digital life.

Digital feedback not only provides material and technological assistance to older adults but also improves their emotional and psychological health status (51). Given older adults' heightened familial dependence and receptivity, digital feedback serves as an intervention to mitigate intergenerational conflicts and enhance family relationships (48). From a family support perspective, such support helps individuals manage stress, anxiety, and related emotional challenges (52). Meanwhile, Li et al. have demonstrated that older adults receive more frequent informational (53), instrumental, and emotional support from younger family members. Such intergenerational support can enhance cognitive functioning and learning motivation while strengthening observational learning effects from digital feedback and reducing technology-related anxiety among older adults (54). Furthermore, rooted in filial piety norms, such support promotes intergenerational harmony while reducing negative affect, amplifying positive emotions, and ultimately enhancing their subjective wellbeing and level of life satisfaction (55).

The above mechanisms lead to the following hypothesis:

H1 Digital feedback has a significant negative effect on health information anxiety in older adults.

### 2.4 Information processing self-efficacy

Bandura, in 1977, first defined self-efficacy as “an individual's speculation and judgment regarding whether they possess the capability to execute specific behaviors (56),” representing the confidence people have in their ability to use their skills to perform a task or behavior rather than actual ability (57). Self-efficacy can be divided into general self-efficacy and specific self-efficacy (58). The former refers to a broad and stable sense of personal competence with universal applicability. Schwarzer et al. developed the General Self-Efficacy Scale in 1981, which has since been translated into at least 25 languages and is widely used internationally (59). The latter connects self-efficacy to particular activity domains (60). For instance, Xie et al. created the College Students' Internet Learning Self-Efficacy Scale based on ternary interaction theory (61) and Anna Zajacova et al. developed the Academic Self-Efficacy Scale through integration and modification of previous studies (62). Regarding older adults, self-efficacy in health information processing belongs to a specific domain. Therefore, introduce the concept of information processing self-efficacy—older adults' speculations and judgments regarding whether they possess the capability to effectively process information through online channels. This conceptualization helps better measure older adults' degree of confidence in searching, filtering, applying, and sharing health information.

Existing studies have primarily examined the influential factors and functional roles of self-efficacy. Bandura's social cognitive theory identifies four primary formation mechanisms of self-efficacy: performance accomplishments, vicarious experience, verbal persuasion, and physiological states (56). When confronting challenges, low self-efficacy individuals typically experience heightened anxiety, are prone to abandonment after failures, and tend to avoid tasks exceeding their perceived capabilities (63). In contrast, high self-efficacy individuals exhibit persistent choice, demonstrating proactive problem-solving, embracing challenges, and firm conviction in goal achievement (64).

### 2.5 Digital feedback and information processing self-efficacy

Older adults are slower to grasp new things, and improper utilization of smart devices and digital networks may compromise their ability to process health information effectively, erode self-confidence, and ultimately lead to digital exclusion (65). Grounded in Bandura's four established pathways of self-efficacy development, the mechanisms through which digital feedback enhances information processing self-efficacy are as follows:

First, positive task experiences enhance individual self-efficacy, while negative experiences produce the opposite effect (66). Research finds that the most significant and direct effect of digital feedback is helping the parental generation solve technical problems (31). Through digital feedback, older adults can acquire enhanced information and communication technology competencies, achieve improved operational efficiency, and obtain superior user experiences—collectively establishing a successful information processing experience that strengthens their self-efficacy (32).

Second, social learning theory indicates that when people in one's social environment routinely use digital devices for health information processing, this behavior can motivate similar adoption among older adults (67). Research confirmed that children's internet application behaviors significantly influence their parents' internet access and usage (7). Meanwhile, digital feedback elevates offspring's perceived competence and social standing in parental perception (68), establishing an effective modeling mechanism. Observational training studies on digital technology use demonstrated significant self-efficacy gains among the post-training older adult (69). Mastering digital competencies also heightens older adults' confidence during peer interactions (20).

Third, people can be successfully convinced to gain confidence in their ability to complete particular tasks or reach predetermined goals (64). During learning technology, older adults who receive guidance, support, and encouragement from their children exhibit more positive attitudes and ensure that they possess the requisite skills and self-assurance (70).

Finally, when individuals comprehend negative emotions and develop an interest in encountered information, this process cultivates self-confidence and self-efficacy (60). Research indicates that digital feedback enhances the frequency of interactions and communication between older adults and their children. The care and recognition from children provide emotional comfort, improve mental health, and ultimately help older adults overcome barriers, strengthening their confidence and motivation to integrate into digital society (67).

Based on Bandura's self-efficacy theory, expectations are categorized into outcome expectations and efficacy expectations (57). Digital feedback strengthens information processing self-efficacy among older adults by acting through four pathways—performance accomplishments, vicarious experience, verbal persuasion, and physiological states—not only reinforcing their belief in the positive outcomes associated with using digital technologies, but also enhancing their confidence in their own ability to effectively process information, thereby strengthening their information processing self-efficacy.

The above mechanisms lead to the following hypothesis:

H2 Digital feedback has a significant positive effect on information processing self-efficacy in older adults.

### 2.6 Information processing self-efficacy and health information anxiety

In addition to expressing a person's belief in their capacity to complete a task, self-efficacy can also influence emotional reactions and indicate anxiety levels (71). When faced with possible threats, calamities, or risks, persons who have high self-efficacy do not feel apprehensive; in contrast, those who have low self-efficacy feel frustrated and anxious about the future because they doubt their capacity to finish tasks and cope with potential dangers (66, 72).

Although the relationship between information processing self-efficacy and health information anxiety has not been fully confirmed, substantial evidence supports the anxiety-reducing effects of self-efficacy, with domain-specific efficacy closely tied to corresponding anxiety types (73). For example, technological innovation self-efficacy is closely related to technological innovation anxiety, where individuals, teams, or organizations with stronger efficacy demonstrate better management of stress, anxiety, and depressive symptoms alongside improved innovation performance (74); academic self-efficacy is closely related to test anxiety, as individuals with higher self-efficacy exhibit lower anxiety levels, greater capacity to adjust learning strategies after failures, and enhanced academic success (75); and computer self-efficacy is closely related to computer anxiety, where heightened efficacy promotes technology acceptance and reduces anxiety (76).

The above mechanisms lead to the following hypothesis:

H3 Information processing self-efficacy has a significant negative effect on health information anxiety in older adults.

### 2.7 The mediating role of information processing self-efficacy

**Figure 1:**
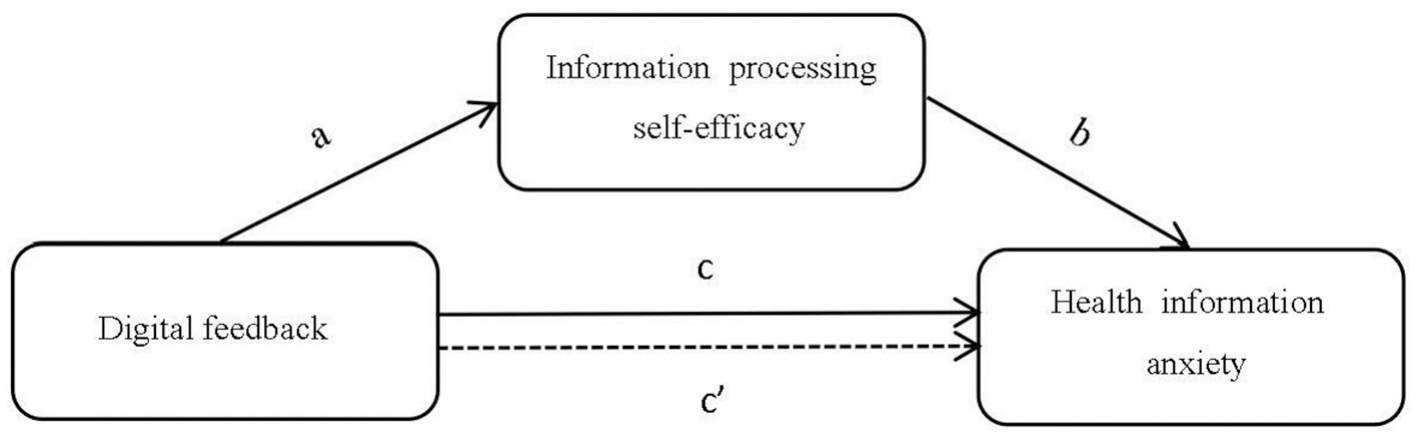
A mediation model of digital feedback influencing health information anxiety.

From the perspective of information ecology theory, older adults' information behaviors are shaped by the interplay of four core elements: the information environment, information technologies, the information itself, and information actors—older adults themselves (77). Digital feedback, as a prototypical form of social support, empowers older adults and facilitates their adaptation to the information ecosystem. On one hand, it provides instrumental and technical support that helps older adults overcome technical barriers, reshape digital cognition, process health information more effectively, and become integrated into the digital environment (78). On the other hand, it offers emotional support by conveying understanding, patience, and care during interpersonal interactions (45). Through digital feedback, the agentic role of older adults as information subjects is strengthened; repeated attempts and successful experiences accumulate positive reinforcement, gradually strengthening their confidence in their own information processing capabilities (79). According to self-efficacy theory, when older adults attain higher levels of information processing self-efficacy, they are more likely to approach challenges with a positive attitude and experience reduced negative emotions in adverse situations (56). This effectively alleviates health information anxiety and enables the transformation of external support into internal psychological resources (Figure 1).

The above mechanisms lead to the following hypothesis:

H4 Information processing self-efficacy mediates the relationship between digital feedback and health information anxiety.
Path a: digital feedback positively influences information processing self-efficacy.
Path b: information processing self-efficacy negatively influences health information anxiety
Path c: digital feedback negatively influences health information anxiety
Path c': information processing self-efficacy mediates the effect of digital feedback on health information anxiety.

## 3 Methodology

### 3.1 Measures

This study invited ten experts from multidisciplinary fields, including management, psychology, sociology, education, and statistics, to conduct two rounds of evaluation on the item content, with a 100% effective response rate achieved in both rounds. The final consultation results showed that the mean importance ratings for all scale items were greater than 3, indicating that the expert panel as a whole endorsed the current evaluation indicator system. Meanwhile, the expert authority coefficient (Cr) was 0.855, suggesting that experts had substantial confidence in their judgments (80). Some experts indicated that the items “digital information organization skill feedback” and “digital information innovation skill feedback” in the digital feedback scale, “access rights anxiety” in the health information anxiety scale, and “perceived control over information processing” in the information processing self-efficacy scale lacked operational feasibility for research on older adults. The coefficient of variation (CV) for these items exceeded 0.25, indicating their removal is warranted. After deletion, the CVs of the remaining items ranged from 0 to 0.22, indicating a high degree of consensus among the experts (81). Finally, based on the expert evaluations, the item-level content validity index (I-CVI) ranged from 0.8 to 1.0, and the average scale-level content validity index (S-CVI/Ave) was 0.93, indicating good content validity (82).

All scale items were subsequently quantified using a standard 7-point Likert scale anchored at 1 = “strongly disagree,” 2 = “disagree,” 3 = “somewhat disagree,” 4 = “neutral,” 5 = “somewhat agree,” 6 = “agree,” and 7 = “very agree.” The specific measurement approach was implemented as follows:

#### 3.1.1 Independent variable: digital feedback

Most scholars divide the digital divide into three progressive levels: the access divide, the usage divide, and the knowledge divide (4, 83). As a key pathway for bridging the digital divide, digital feedback is interdependent and mutually constraining with the digital divide, leading to a natural correspondence in their dimensional structure. Based on this, scholar Zhou and Ding categorizes digital feedback into three dimensions: digital access feedback, digital skill feedback, and digital literacy feedback (19). This framework not only aligns well with instrumental and informational support within social support theory but also implicitly includes emotional support (84), reflecting a dynamic process through which younger generations' support for older generations extends from concrete behaviors to cognitive and conceptual change, and from technology use to shifts in thinking patterns (85). Subsequent studies have largely adopted this three-dimensional classification (19, 86).

Digital access feedback: children facilitate older adults' access to digital devices and internet connectivity, enabling a digital environment.

Digital skill feedback: children assist older adults in acquiring operational competencies for digital devices (this specifically refers to older adults requiring the ability to process information).

Digital literacy feedback: children impart digital knowledge and experience to older adults, enhancing their comprehension of digital technologies and adherence to cyberspace norms. This dimension includes higher-order value norms as well as basic attitudes and beliefs about digital technologies (87).

As presented in Table 1, the finalized scale comprises 11 items, with higher scores indicating stronger digital feedback engagement. Cronbach's α of the scale was 0.841, indicating good reliability.

**Table 1:** Digital feedback scale.

**First-level dimension**	**Second-level dimensions**	**Third-level dimensions**	**Questions**
Digital feedback	Digital access feedback	a1 Network access feedback	Your children help you establish internet connectivity.
		a2 Hardware access feedback	Your children help you acquire digital devices (e.g., smartphones).
		a3 Software access feedback	Your children help you download and install applications.
	Digital skill feedback	a4 Digital information retrieval skill feedback	Your children teach you to master searching and obtaining information skills through multiple channels(e.g., search engines, WeChat official accounts).
		a5 Digital information filtering skill feedback	Your children teach you to master evaluating and selecting high-quality content skills.
		a6 Digital information application skill feedback	Your children teach you to master information application skills for problem-solving.
		a7 Digital information interaction skill feedback	Your children teach you to master digital information transmission, sharing, and social interaction skills through digital platforms (e.g., WeChat, Weibo, Douyin).
	Digital literacy feedback	a8 Digital willingness feedback	Your children encourage your participation in digital environments and enhance your willingness to exposure and adopt technologies.
		a9 Digital cognitive feedback	Your children explain the significance, life impacts, and future development of digital technologies to deepen your awareness and understanding.
		a10 Digital security feedback	Your children share your knowledge about cybersecurity risks (e.g., online fraud, privacy protection).
		a11 Digital ethical feedback	Your children guide you in adopting appropriate digital ethics principles and behavioral norms (e.g., respecting privacy, safeguarding intellectual property rights).

#### 3.1.2 Dependent variable: health information anxiety

There are two predominant approaches in academia for measuring information anxiety: the first assesses anxiety separately across different stages of information processing, with the composite score representing overall information anxiety; the second evaluates anxiety based on distinct causative factors (64). This study adopts the latter approach and integrates information ecology theory to dimensionalize health information anxiety among older adults.

According to the theory, the information ecosystem is an organic whole made up of four ecological factors that are interconnected and dynamically interact with one another: informants, information, information technology, and information environment (77, 88). In this study, the informant refers to older adult health information users, the information denotes online health information, the information technology represents health information platforms, and the information environment captures the environmental setting where older adult users search, share, and utilize health information. These collectively constitute the determinants of health information anxiety among older adults, which can be classified into four distinct dimensions: self-perceived anxiety, information-driven anxiety, technology-induced anxiety, and environment-triggered anxiety.

Self-perceived anxiety: anxiety arising from self-evaluation and subjective cognition during health information processing among older adults.

Information-driven anxiety: anxiety arising from information-related issues such as information quality or information alienation among older adults.

Technology-induced anxiety: anxiety arising from technical barriers of platforms when using health information platforms among older adults.

Environment-triggered anxiety: anxiety arising from the online environment during health information processing among older adults.

As presented in Table 2, the finalized scale comprises 16 items, with higher scores indicating greater severity of health information anxiety. Cronbach's α of the scale was 0.779, indicating good reliability.

**Table 2:** Health information anxiety scale.

**First-level dimension**	**Second-level dimensions**	**Third-level dimensions**	**Questions**
Health information anxiety	Self-perceived anxiety	c1 Perceived information risk anxiety	You often misinterpret or exaggerate health information, feeling that your own or your family's health is at risk
		c2 Perceived information craving anxiety	You often browse health information uncontrollably and constantly refresh to obtain satisfactory information
		c3 Perceived information missing anxiety	You often feel anxious about missing health information that others may have received
		c4 Perceived information time-cost anxiety	You often spend too much time on health information and feel guilty and distressed as a result
	Information-driven anxiety	c5 Information false anxiety	You worry about whether the health information you obtain is authentic
		c6 Information ambiguity anxiety	You are concerned that the health information you access is unclear or difficult to understand
		c7 Information conflict anxiety	You worry about contradictions or conflicts in health information from different sources
		c8 Information overload anxiety	You are concerned that the amount of health information exceeds your ability to receive and process it
		c9 Information cocoon anxiety	You worry about being misled by homogeneous health information pushed by platforms
	Technology-induced anxiety	c10 System stability anxiety	When accessing health information platforms, you feel disappointed and frustrated if information fails to load, links break, or network errors occur
		c11 System compatibility anxiety	When health information platforms are incompatible, you feel at a loss
		c12 Interface adaptability anxiety	When platform interfaces (e.g., fonts, colors, layouts) are overly complex or disordered and incompatible with your visual and operational habits, you feel annoyed and impatient
		c13 Function completeness anxiety	When health information platforms fail to meet your needs in terms of functions and services, you feel worried
	Environment-triggered anxiety	c14 Regulatory environment anxiety	You often worry about ineffective online regulation leading to personal information leakage
		c15 Social environment anxiety	You often worry about facing discrimination or exclusion when sharing health information or expressing health-related opinions
		c16 Public opinion environment anxiety	You often worry that health information may be exaggerated or distorted during dissemination, causing public opinion to deviate from facts

#### 3.1.3 Mediating variable: information processing self-efficacy

Individuals with varying self-efficacy levels select tasks of differing difficulty (64). Those with high self-efficacy generally demonstrate stronger motivation, greater willingness to expend effort, and more proactive engagement in problem-solving (89). Building upon this study's framework and the definition of self-efficacy, information processing self-efficacy is categorized into three dimensions: information processing adaptation perception, information processing effort perception, and information processing competence perception, mapping onto the distinct stages and levels of psychological changes during health information processing in older adults. This delineation captures older adults' progressive self-recognition of their information processing competence, accompanied by heightened self-confidence and reinforced motivational levels.

Information processing adaptation perception: older adults' speculation and judgment of whether they can preliminarily adapt to digital environments and acquire information through online platforms.

Information processing effort perception: older adults' speculation and judgment of whether they can progressively enhance information-processing abilities through sustained effort.

Information processing competence perception: older adults' speculation and judgment of whether they possess the necessary skills and knowledge to complete specific information processing tasks.

As presented in Table 3, the finalized scale comprises 16 items, with higher scores indicating stronger information processing self-efficacy. Cronbach's α of the scale was 0.843, indicating good reliability.

**Table 3:** Information processing self-efficacy scale.

	**Dimensions**	**Questions**
Information processing self-efficacy	Information processing adaptation perception	b1 You habitually use the internet as a primary information acquisition channel
		b2 You can quickly familiarize yourself with various information platforms (e.g., websites, official accounts, application software)
		b3 You adapt to using different search methods (e.g., keyword search, voice search, image recognition search) to obtain information
	Information processing effort perception	b4 You have exerted substantial effort to acquire useful information
		b5 You have invested considerable effort to resolve difficulties encountered during information processing
		b6 You have devoted significant effort to improving information processing efficiency
	Information processing competence perception	b7 You can effortlessly access needed information
		b8 You can make correct decisions based on obtained information
		b9 When encountering information processing difficulties, you can generate multiple solutions
		b10 You possess more effective information than others
		b11 The information you share benefits others

#### 3.1.4 Control variables

To enhance the precision of our analysis, gender (90), age group (91), educational level (31), number of children (7), residence area (32), living arrangement (92), economic status (93), and health status (43) were included as control variables, consistent with established research.

### 3.2 Procedures and participants

First, a preliminary survey was administered to older adults, with 100 questionnaires distributed and 86 valid responses collected. Then, initial reliability and validity analyses were conducted, which indicated the basic rationality of the questionnaire design. Finally, imprecise, unclear, or ambiguous items were refined through expert recommendations to enhance the instrument's validity. The additional document shows the questionnaire in detail (see Supplementary File S1).

This study focuses on older adults in China, with the official survey period spanning from June 2024 to February 2025, conducted through four sequential stages: first, 3–6 cities were randomly selected from North China, East China, Northeast China, Central China, South China, Northwest China, and Southwest China. The final city sample included two directly administered municipalities (Beijing and Shanghai), six provincial capitals (Shijiazhuang, Nanjing, Shenyang, Wuhan, Guangzhou, and Lanzhou), and 22 prefecture-level cities (Hengshui, Datong, Changzhou, Shaoxing, Wuhu, Quanzhou, Yingkou, Luoyang, Xiangyang, Changde, Foshan, Liuzhou, Wuzhou, Baoji, Tianshui, Shizuishan, Karamay, Mianyang, Zunyi, Yuxi, Leshan, and Liupanshui). Second, the sampling process utilized proportional to size (PPS) methodology (94), selecting two districts or counties from each city (or directly sampling streets/townships for cities with limited administrative divisions). Then, within each district or county, three streets/townships were selected based on economic stratification (good, medium, and poor levels). Next, from each street/township, one neighborhood/village committee was selected. Finally, 10–12 households were selected from each neighborhood or village committee, where surveys were conducted if older adult individuals were present. If none were available, the process proceeded to adjacent households, ensuring a minimum of 10 participants per neighborhood or village committee while maintaining approximate gender parity. The study ultimately included 1,900 older adult individuals.

Considering the distinctive characteristics of older adults, the study employed a dual-mode survey approach combining self-administered and proxy-administered questionnaires, delivered through both online and offline channels. The online component utilized questionnaires created via the Questionnaire Star platform, disseminated through mainstream social media platforms (e.g., WeChat and Weibo). The offline questionnaires were distributed by investigators who visited survey respondents' homes or conducted outreach at senior activity rooms, parks, and other locations frequently visited by older adults. Investigators followed a standardized procedure of explaining the survey purpose to survey respondents, obtaining their consent, and providing clarification for any questions that required explanation. Additionally, for older adults who were unable to complete the questionnaire independently due to physical or technical limitations, investigators transcribed the questionnaire responses based on older adults' oral answers to ensure the reliability and authenticity of the questionnaire data. To qualify for inclusion, eligible survey respondents in this study were required to meet all four criteria: being 60 years of age or older with intact cognitive function and capacity for independent critical thinking; having at least one child; having engaged in health information-seeking behaviors within the past year; and voluntarily agreeing to participate in the survey by providing signed informed consent.

**Figure 2:**
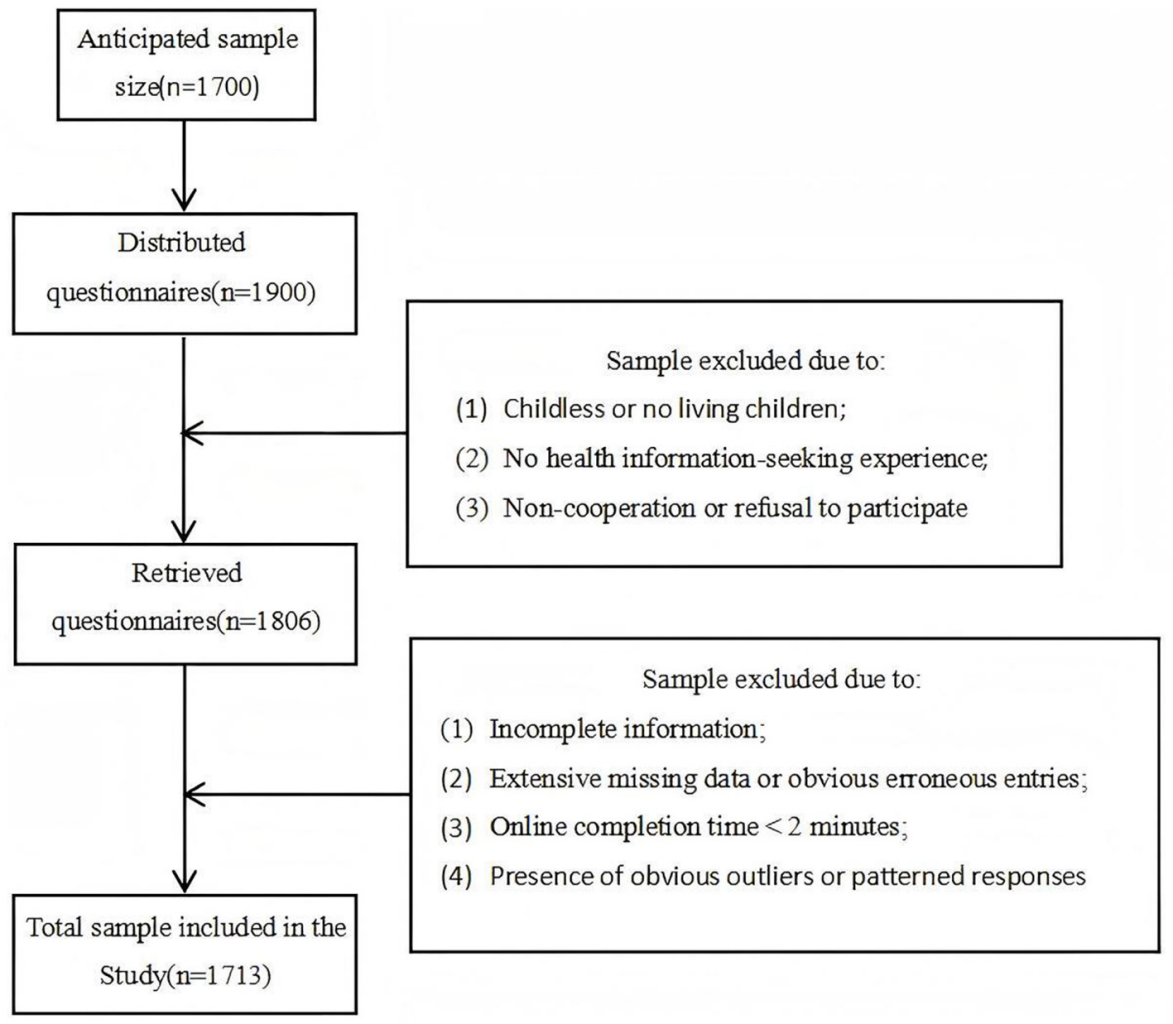
Flowchart of survey respondents selection process.

A total of 1,900 questionnaires were distributed in this study. After excluding respondents who had no children or whose children had passed away, those with no experience in health information searching, and those who were uncooperative or refused to participate, 1,806 questionnaires were collected. Following rigorous manual review and screening, additional questionnaires were excluded due to incomplete responses, extensive missing data, obvious response errors, online completion time of less than 2 min, or presence of significant outliers or patterned responding. Ultimately, 1,713 valid questionnaires were retained, yielding an effective response rate of 90.16% (Figure 2). Previous studies suggest that for exploratory factor analysis (EFA), the sample size should be at least 10 times the number of items in the longest scale, with 20 times being preferable. The longest scale in this study contains 16 items; therefore, the sample size in this study far exceeds the minimum requirement and is sufficient to meet the needs of model estimation (95).

### 3.3 Sample description

The sample characteristics are presented in Table 4.

**Table 4:** Demographic characteristics of the sample.

**Variable**	**Category**	***n***	**%**	**Variable**	**Category**	***n***	**%**
Gender	Male	846	49.39	Living arrangement	Living alone	247	14.42
	Female	867	50.61		With spouse only	725	42.32
Age group (years)	60–69	918	53.59		With children only	273	15.94
	70–79	514	30.01		With spouse and children	357	20.84
	≥80	281	16.4		With other relatives	111	6.48
Education level	Primary or below	958	55.93	Economic status	Very poor	129	7.53
	Junior high	407	23.76		Poor	284	16.58
	Specialized secondary/senior high	221	12.90		Average	548	31.99
	Junior college or above	127	7.41		Good	635	37.07
Number of children	1	669	39.05		Excellent	117	6.83
	2	514	30.01	Health status	Very poor	145	8.46
	3	381	22.24		Poor	229	13.37
	≥4	149	8.70		Average	656	38.30
Residence area	Rural	808	47.17		Good	456	26.62
	Urban	905	52.83		Excellent	227	13.25

### 3.4 Date analysis

Data analysis was performed using SPSS 27.0. First, scale validation was performed through exploratory factor analysis. Second, common method bias was tested using Harman's single-factor test. Then, descriptive statistical analysis and Pearson correlation analysis were performed for all variables. Next, the causal steps approach was employed to preliminarily examine the mediating role of information processing self-efficacy between digital feedback and health information anxiety. Finally, mediation analysis was performed using PROCESS v4.1 in SPSS to validate the hypotheses further.

### 3.5 In-depth interviews

Based on a questionnaire search, five older adult individuals who regularly use the Internet for health information processing were selected for semi-structured interviews to investigate the mechanisms through which digital feedback influences health information anxiety. The interviews focused on perspectives regarding digital technology, experiences during digital feedback interactions, and challenges in health information processing, among other relevant aspects. All interview participants volunteered for the study, with each interview lasting over 30 min on average. With the interview participants' consent, all interviews were audio-recorded for subsequent verbatim transcription. Any unclear content was clarified through follow-up contact with the interview participants. The additional document shows the interview outline in detail (see Supplementary File S2).

The total interview duration for the five participants was approximately 189 min, and all audio recordings were transcribed verbatim, resulting in approximately 32,000 words of textual data. To deeply explore the interview data, this study referred to Braun and Clarke's thematic analysis approach to code the interview transcripts (96). First, the research team became familiar with the text through repeated reading and conducted line-by-line coding of the text, generating a large number of initial codes such as “purchasing digital devices,” “actively exploring and learning,” and “not understanding information content.” Subsequently, through continuous comparison, categorization, and integration of the initial codes, six overarching themes were identified: “practices of digital feedback,” “experiences of digital feedback,” “development of information processing self-efficacy,” “sources of health information anxiety,” “manifestations of health information anxiety,” and “alleviation of health information anxiety.” All themes centered on the core theme of “the impact of digital feedback on health information anxiety,” as shown in Table 5.

**Table 5:** Results of thematic analysis.

**Core theme**	**Main themes**	**Initial codes**		**Frequency**
		**Sub-themes**	**Examples**	
The impact of digital feedback on health information anxiety	Practices of digital feedback	Feedback contexts	Active help-seeking; passive learning	5
		Feedback frequency	Daily; weekly; monthly; during holidays	5
		Feedback content	Purchasing digital devices; installing software; searching for information; filtering information; sharing information; teaching digital knowledge	14
	Experiences of digital feedback	Positive experiences	Happiness; reassurance; sense of achievement; satisfaction; feeling cared for	9
		Negative experiences	Boredom; impatience	3
	Development of information processing self-efficacy	Emotional manifestations	Confidence; self-identity; sense of control	6
		Behavioral manifestations	Actively exploring and learning; problem-solving orientation; trying new features; helping peers; sharing new discoveries	16
	Sources of health information anxiety	Information characteristics	Contradictory information; false information; exaggerated information; information overload	12
		Personal competence	Unable to connect to the internet; unable to operate devices; inability to identify valid information; difficulty understanding content	15
		Environmental pressures	Information leakage; online fraud; internet rumors; social risks	12
		Platform deficiencies	Font too small; cluttered interface; system lag	4
	Manifestations of health information anxiety	Emotional manifestations	Persistent worry; irritability; fear; tension; helplessness	18
		Physiological and behavioral manifestations	Headache; insomnia; difficulty concentrating; repeated searching; giving up searching	11
	Alleviation of health information anxiety	Emotional improvement	Relaxed mindset; reduced stress; feeling reassured	14
		Behavioral optimization	More precise searching; rational evaluation; improved efficiency	10

## 4 Results

### 4.1 Exploratory factor analysis

Exploratory factor analysis (EFA) and confirmatory factor analysis (CFA) constitute distinct methodological approaches for assessing scale structural validity. EFA is employed to identify latent structural dimensions through data analysis when clear theoretical expectations are absent, particularly suited for newly developed or insufficiently validated scales. In contrast, CFA tests the alignment between observed variables and a theoretically predefined factor structure under existing theoretical frameworks to verify its conceptual soundness and measurement adequacy (97). Since this study utilized an original scale, EFA was conducted to assess whether the derived factor structure corresponded to the hypothesized dimensional framework.

The results showed that the scale had a KMO value of 0.938, and Bartlett's test of sphericity was significant (*p* < 0.001), indicating that all items were suitable for factor analysis. Based on the factor loading coefficient matrix, factor 1 (items a1–a3) had eigenvalue = 12.275 with loadings = 0.718–0.834, and accounted for 32.304% of the total variance; factor 2 (items a4–a7) had eigenvalue = 2.788 with loadings = 0.693–0.755, and accounted for 39.641% of the total variance; factor 3 (items a8–a11) had eigenvalue = 1.877 with loadings = 0.699–0.753, and accounted for 44.579% of the total variance; factor 4 (items b1–b3) had eigenvalue = 1.800 with loadings = 0.778–0.821, and accounted for 49.315% of the total variance; factor 5 (items b4–b6) had eigenvalue = 1.761 with loadings = 0.755–0.804, and accounted for 53.95% of the total variance; factor 6 (items b7–b11) had eigenvalue = 1.600 with loadings = 0.670–0.751, and accounted for 58.16% of the total variance; factor 7 (items c1–c4) had eigenvalue = 1.423 with loadings = 0.835–0.851, and accounted for 61.904% of the total variance; factor 8 (items c5–c9) had eigenvalue = 1.334 with loadings = 0.667–0.734, and accounted for 65.415% of the total variance; factor 9 (items c10–c13) had eigenvalue = 1.083 with loadings = 0.762–0.795, and accounted for 68.265% of the total variance; factor 10 (items c14–c16) had eigenvalue = 1.013 with loadings = 0.708–0.807, and accounted for 70.93% of the total variance. The final factor structure derived from the factor loading analysis was consistent with the original dimensional framework.

The composite reliability (CR) of the digital feedback scale was calculated as 0.93, with an average variance extracted (AVE) of 0.53; the information processing self-efficacy scale yielded a CR of 0.93 and an AVE of 0.59; and the health information anxiety scale demonstrated a CR of 0.96 and an AVE of 0.6, indicating that all scales exhibit high reliability and strong explanatory power (98).

### 4.2 Common method bias

The Harman single-factor test has relatively low statistical power and is highly sensitive to the number of measured constructs and scale reliability; however, due to its convenience, it remains widely used in empirical research (99). This study also employed Harman's single-factor test to assess common method bias. The results of unrotated principal component analysis revealed that 10 factors had initial eigenvalues greater than 1, cumulatively accounting for 70.920% of the total variance, demonstrating satisfactory explanatory power for the original variables. Notably, the first factor explained 32.299% of the variance, which was below the critical threshold of 40%, indicating no significant common method bias in the study.

### 4.3 Descriptive statistics and correlation analyses

As shown in Table 6, the mean scores of “digital feedback,” “information processing self-efficacy,” and “health information anxiety” among older adults were all slightly above the average level (with a midpoint of 4 on the 7-point Likert scale used in this study). Pearson correlation analysis revealed significant positive correlations between digital feedback and information processing self-efficacy (*r* = 0.717, *p* < 0.01), significant negative correlations between digital feedback and health information anxiety (*r* = −0.550, *p* < 0.01), and significant negative correlations between information processing self-efficacy and health information anxiety (*r* = −0.669, *p* < 0.01) among older adults. These findings support the testing of the research hypotheses.

**Table 6:** Results of means, standard deviations, and correlations.

**Variable**	**1**	**2**	**3**	**4**	**5**	**6**	**7**	**8**	**9**	**10**	**11**	**12**	**13**	**M**	**SD**
1. Digital feedback	1													4.35	1.03
2. Digital access feedback	0.719^**^	1												4.55	1.34
3. Digital skill feedback	0.762^**^	0.373^**^	1											4.33	1.29
4. Digital literacy feedback	0.794^**^	0.386^**^	0.345^**^	1										4.23	1.41
5. Information processing self-efficacy	0.717^**^	0.438^**^	0.618^**^	0.558^**^	1									4.16	1.10
6. Information processing adaptation perception	0.551^**^	0.399^**^	0.465^**^	0.393^**^	0.716^**^	1								4.16	1.64
7. Information processing effort perception	0.508^**^	0.298^**^	0.426^**^	0.416^**^	0.653^**^	0.270^**^	1							4.27	1.50
8. Information processing competence perception	0.546^**^	0.297^**^	0.485^**^	0.438^**^	0.830^**^	0.376^**^	0.315^**^	1						4.09	1.36
9. Health information anxiety	−0.550^**^	−0.252^**^	−0.488^**^	−0.475^**^	−0.669^**^	−0.452^**^	−0.460^**^	−0.554^**^	1					4.22	0.86
10. Self–perceived anxiety	0.433^**^	0.344^**^	0.322^**^	0.328^**^	0.251^**^	0.204^**^	0.187^**^	0.183^**^	0.134^**^	1				4.31	1.63
11. Information–driven anxiety	−0.644^**^	−0.353^**^	−0.552^**^	−0.534^**^	−0.668^**^	−0.465^**^	−0.470^**^	−0.541^**^	0.762^**^	−0.304^**^	1			4.19	1.42
12. Technology–induced anxiety	−0.538^**^	−0.297^**^	−0.477^**^	−0.430^**^	−0.573^**^	−0.396^**^	−0.393^**^	−0.470^**^	0.731^**^	−0.232^**^	0.507^**^	1		4.16	1.54
13. Environment-triggered anxiety	−0.553^**^	−0.305^**^	−0.434^**^	−0.494^**^	−0.570^**^	−0.402^**^	−0.397^**^	−0.462^**^	0.661^**^	−0.238^**^	0.503^**^	0.409^**^	1	4.24	1.54

^**^p < 0.01.

Additionally, given the relatively high correlation coefficients observed between some variables, a multicollinearity test was conducted. The results showed that all variance inflation factor values ranged from 1.205 to 1.564, indicating low risk of multicollinearity and good model stability (100).

### 4.4 Causal steps approach

Following the principles of mediation analysis, testing whether variable M mediates the relationship between variables X and Y must be conducted before performing model regression. The most commonly used method is the causal steps approach (Table 7) (101–103), which offers straightforward implementation and broad applicability while demonstrating both direct and indirect relationships between variables for easier understanding and interpretation. Additionally, this method effectively addresses multicollinearity issues, improves test robustness, and minimizes false results.

**Table 7:** Results of causal steps approach (dimensional analysis).

**Variable**	**Health information anxiety**																**Information processing self-efficacy**		
	**Self-perceived anxiety**				**Information-driven anxiety**				**Technology-induced anxiety**				**Environment-triggered anxiety**				**Information processing adaptation perception**	**Information processing effort perception**	**Information processing competence perception**
	**(1)**	**(2)**	**(3)**	**(4)**	**(5)**	**(6)**	**(7)**	**(8)**	**(9)**	**(10)**	**(11)**	**(12)**	**(13)**	**(14)**	**(15)**	**(16)**	**(17)**	**(18)**	**(19)**
**Digital feedback**																			
Digital access feedback	0.265^***^	0.274^***^	0.267^***^	0.267^***^	−0.039	−0.014	−0.030	−0.033	−0.034	−0.012	−0.027	−0.028	−0.044	−0.016	−0.036	−0.038	0.223^***^	0.066^*^	0.029
Digital skill feedback	0.245^***^	0.261^***^	0.255^***^	0.270^***^	−0.403^***^	−0.360^***^	−0.359^***^	−0.325^**^	−0.379^***^	−0.341^***^	−0.341^***^	−0.303^***^	−0.315^***^	−0.268^***^	−0.273^***^	−0.237^***^	0.374^***^	0.318^***^	0.351^***^
Digital literacy feedback	0.224^***^	0.233^***^	0.233^***^	0.242^***^	−0.348^***^	−0.323^***^	−0.309^***^	−0.290^***^	−0.278^***^	−0.256^***^	−0.245^***^	−0.222^***^	−0.394^***^	−0.366^***^	−0.357^***^	−0.336^***^	0.219^***^	0.281^***^	0.260^***^
**Information processing self-efficacy**																			
Information processing adaptation perception		−0.043				−0.114^***^				−0.102^***^				−0.126^***^					
Information processing effort perception			−0.032				−0.140^***^				−0.119^***^				−0.132^***^				
Information processing competence perception				−0.071				−0.223^***^				−0.218^***^				−0.223^***^			
*R* ^2^	0.202	0.203	0.202	0.204	0.473	0.484	0.488	0.502	0.348	0.356	0.358	0.372	0.336	0.345	0.348	0.362	0.326	0.283	0.344
Adj *R*^2^	0.196	0.197	0.197	0.198	0.469	0.481	0.484	0.499	0.344	0.351	0.353	0.368	0.332	0.341	0.344	0.357	0.321	0.278	0.340
*F*	39.048^***^	36.050^***^	35.914^***^	36.286^***^	138.517^***^	133.038^***^	135.069^***^	143.079^***^	82.493^***^	78.283^***^	78.840^***^	83.989^***^	78.374^***^	74.766^***^	75.680^***^	80.297^***^	74.743^***^	60.995^***^	81.123^***^

^*^p < 0.05, ^**^p < 0.01, ^***^p < 0.001.

First, regression analyses were conducted using the mean scores of the three dimensions of digital feedback as independent variables and the mean scores of the four dimensions of health information anxiety as dependent variables. Model 1 (the coefficients were 0.265, 0.245, 0.224, respectively) demonstrated that all three dimensions of digital feedback had significant positive effects on self-perceived anxiety. Similarly, Models 5 (the coefficients were −0.039, −0.403, −0.348, respectively), 9 (the coefficients were −0.034, −0.379, −0.278, respectively), and 13 (the coefficients were −0.044, −0.315, −0.394, respectively) indicated that digital skills feedback and digital literacy feedback exerted significant negative effects on information-driven anxiety, technology-induced anxiety, and environment-triggered anxiety, whereas digital access feedback showed no significant effects on these three dimensions.

Second, regression analyses were conducted using the mean scores of the three dimensions of digital feedback as independent variables and the mean scores of the three dimensions of information processing self-efficacy as dependent variables. Models 17 (the coefficients were 0.223, 0.374, 0.219, respectively) and 18 (the coefficients were 0.066, 0.318, 0.281, respectively) demonstrated that all three dimensions of digital feedback had significant positive effects on both information processing adaptation perception and information processing effort perception. Model 19 (the coefficients were 0.029, 0.351, 0.260, respectively) revealed that only digital skill feedback and digital literacy feedback showed significant positive effects on information processing competence perception.

Third, regression analyses were conducted by separately introducing the mean scores of the three dimensions of information processing self-efficacy as mediator variables, the mean scores of the three dimensions of digital feedback as independent variables, and the mean scores of the four dimensions of health information anxiety as dependent variables. The results showed that all three dimensions of digital feedback maintained significant positive effects on self-perceived anxiety, while none of the three dimensions of information processing self-efficacy showed significant effects on self-perceived anxiety; both digital skill feedback and digital literacy feedback continued to demonstrate significant negative effects on information-driven anxiety, technology-induced anxiety, and environment-triggered anxiety; meanwhile, all three dimensions of information processing self-efficacy also exhibited significant negative effects on information-driven anxiety, technology-induced anxiety, and environment-triggered anxiety. These findings suggest that information processing self-efficacy partially mediates the effect of digital feedback on health information anxiety.

To further examine the global effects of digital feedback on health information anxiety and the mediating role of information processing self-efficacy, a global analysis was performed using the scale's total mean scores (Table 8). (1) With the mean score of digital feedback as the independent variable and the mean score of health information anxiety as the dependent variable, results showed that digital feedback negatively affected health information anxiety in older adults (β = −0.396, *p* < 0.001), confirming Hypothesis 1. Moreover, older adults with higher age and lower education levels showed higher levels of health information anxiety. (2) With the mean score of digital feedback as the independent variable and the mean score of information processing self-efficacy as the dependent variable, results showed that digital feedback positively affected information processing self-efficacy in older adults (β = 0.700, *p* < 0.001), confirming Hypothesis 2. Moreover, older adults with younger ages and higher education levels showed higher information processing self-efficacy. (3) With the mean score of digital feedback as the independent variable, the mean score of information processing self-efficacy as the mediator variable, and the mean score of health information anxiety as the dependent variable, results showed that digital feedback still negatively affected health information anxiety (β = −0.116, *p* < 0.001), and information processing self-efficacy also negatively affected health information anxiety (β = −0.401, *p* < 0.001), confirming Hypothesis 3. (4) |β| for digital feedback decreased from 0.396 in Model 1 to 0.116 in Model 3, reaching statistical significance, showing that information processing self-efficacy partially mediated the effect of digital feedback on health information anxiety in older adults, confirming Hypothesis 4.

**Table 8:** Results of causal steps approach (global analysis).

**Variable**	**Health information anxiety**	**Information processing self-efficacy**	**Health information anxiety**
	**(1)**	**(2)**	**(3)**
Digital feedback	−0.396^***^	0.700^***^	−0.116^***^
Information processing self-efficacy			−0.401^***^
Gender	−0.038	0.009	−0.034
Age group	0.204^***^	−0.196^***^	0.125^***^
Education level	−0.152^***^	0.142^***^	−0.095^***^
Number of children	−0.029	0.005	−0.027
Residence area	0.012	−0.017	0.005
Living arrangement	−0.005	0.026	0.005
Economic status	0.021	0.003	0.022
Health status	−0.021	0.007	−0.018
*R* ^2^	0.360	0.547	0.479
Adj *R*^2^	0.357	0.545	0.476
*F*	106.550^***^	228.783^***^	156.298^***^

^***^p < 0.001.

### 4.5 Mediation analysis

Some scholars have noted that Baron and Kenny's causal steps approach lacks a unified framework for overall model evaluation, increases the risk of multiple comparisons, and demonstrates insufficient power when testing weak mediation effects. Therefore, this study further employed the more widely recommended bootstrap method to examine mediation effects (104–107). The bootstrap method, based on the theoretical concept of standard errors, uses resampling techniques to estimate effects by repeatedly drawing subsamples with replacement from the original sample. This approach does not rely on normality assumptions and yields more accurate test results when sufficient resampling iterations are performed.

The study used the PROCESS macro model 4 with 5,000 bootstrap resamples for analysis. As shown in Table 9, the 95% confidence intervals for all path coefficients excluded zero, indicating statistically significant mediation effects. Digital feedback negatively predicted health information anxiety (β = −0.3962, SE = 0.0179). After introducing information processing self-efficacy as a mediator, digital feedback remained a significant predictor of health information anxiety (β = −0.1156, SE = 0.0216). Concurrently, digital feedback positively predicted information processing self-efficacy (β = 0.6996, SE = 0.0192), while information processing self-efficacy negatively predicted health information anxiety (β = −0.4011, SE = 0.0204), consistent with the hypotheses. Furthermore, information processing self-efficacy exhibited a significant indirect effect (β = −0.2806, SE = 0.0157). These results collectively indicate that information processing self-efficacy partially mediates the relationship between digital feedback and health information anxiety.

**Table 9:** Results of mediation analysis.

**Effect**	**β**	**SE**	**95.0% confidence interval**	
			**LLCI**	**ULCI**
**Direct effect**				
Digital feedback—information processing self-efficacy	0.6996	0.0192	0.6619	0.7373
Information processing self-efficacy—health information anxiety	−0.4011	0.0204	−0.4411	−0.3611
Digital feedback—health information anxiety	−0.1156	0.0216	−0.1579	−0.0733
**Indirect effect**				
Digital feedback—information processing self-efficacy—health information anxiety	−0.2806	0.0157	−0.3115	−0.2503
**Total effect**				
Digital feedback—information processing self-efficacy—health information anxiety	−0.3962	0.0179	−0.4313	−0.3610

LLCI, lower limit of confidence interval; ULCI, upper limit of confidence interval.

These findings indicate that information processing self-efficacy partially mediates the relationship between digital feedback and health information anxiety, with a mediation effect accounting for 69.88%. This suggests that enhancing older adults' information processing self-efficacy through digital feedback is a key pathway to alleviating their health information anxiety. Therefore, in addition to continuously optimizing the information environment, practical interventions should focus on psychological empowerment for older adults, fully leveraging familial support. As primary providers of digital feedback, children can set learning goals for older adults and employ progressive guidance, ongoing encouragement, and feedback on successful experiences to help them build confidence and self-identity through real-world health information processing tasks, thereby reducing anxiety.

## 5 Discussion

### 5.1 The alleviating effect of digital feedback on health information anxiety among older adults

The findings support Hypothesis 1, confirming that digital feedback effectively alleviates health information anxiety among older adults, which aligns with most previous research (30, 50, 108). The findings revealed that digital skills feedback demonstrates superior efficacy in alleviating information-driven anxiety and technology-induced anxiety compared to digital access feedback and digital literacy feedback.

“I saw something online about ‘miracle cures'; my daughter immediately checked it for me and confirmed it was fake. Then she recommended some official websites to me, so now I don't worry about false information anymore.” (Interview A) “My children taught me how to set up ‘older adult' mode—the text is bigger now so I don't have to worry about not being able to read it.” (Interview B)

Technical challenges constitute the primary barrier for older adults when processing online health information. Digital skill feedback enhances their capacity to search, comprehend, and evaluate health information while also fostering their active participation in health information exchanges and interactions to solve practical problems, thereby reducing anxiety (109). Additionally, digital literacy feedback demonstrated superior efficacy in alleviating environment-triggered anxiety compared to digital access feedback and digital skill feedback.

“I saw news reports about people losing money from their accounts after clicking wrong links, which made me concerned about the online security environment. Later, my daughter taught me how to identify phishing ads, and now I feel more secure when going online.” (Interview C)

Through digital literacy feedback, older adults develop a deeper understanding of digital information and technologies, enabling them to better identify online risks and master normative behavioral standards in cyberspace, thereby facilitating their adaptation to the online health information environment (67, 110).

Notably, digital feedback may exacerbate self-perceived anxiety—a phenomenon documented in prior literature. If children neglect offline communication and companionship with their parents, some older adults may, after acquiring basic digital skills, become overly reliant on information obtained online (111), frequently refreshing feeds or conducting repeated searches to alleviate inner loneliness and anxiety (112). At the same time, they are prone to misinterpret or over-interpret information, leading to excessive concerns about their own and their family's health, resulting in sustained risk vigilance and psychological burden—facing a vicious cycle of “the more they search, the more anxious they become (40).”

### 5.2 The enhancing effect of digital feedback on information processing self-efficacy among older adults

The findings support Hypothesis 2, confirming that digital feedback effectively enhances information processing self-efficacy among older adults. The findings revealed that both digital skills feedback and digital literacy feedback demonstrate stronger correlations with information processing self-efficacy compared to digital access feedback.

“My son taught me how to search for short videos about hypertension, now I can even share and comment on them” (Interview D). “My son told me never to casually enter sensitive information like home addresses or ID numbers online. Now, I'm much more cautious—when I'm unsure about a website, I forward it to my children. I've learned to protect myself online.” (Interview E).

The internet usage experience transferred from children not only strengthens older adults' confidence in dealing with online health information but also helps them gain a sense of accomplishment in addressing health-related issues, motivating them to continue exploring and utilizing digital technologies to improve their quality of life (79). Furthermore, a substantial body of research demonstrateed that digital feedback significantly improves intergenerational relationships (20, 113). Children offer companionship to their parents through digital feedback, and the parents perceive their children's support and care, which helps them gain confidence to integrate into the digital world.

“My children taught me step by step, and now using electronic devices doesn't seem so difficult anymore—it has also brought me closer to them.” (Interview F)

Notably, some scholars have also pointed out that not all instances of digital feedback are successful (53, 68). This may be attributed to insufficient depth or impatience in children's instruction, as well as low receptiveness or difficulties in adapting to shifts in parental authority among older adults. Not only might inappropriate methods of providing digital feedback reduce its usefulness, but they may also cause older adults' self-efficacy to diminish.

### 5.3 The alleviating effect of information processing self-efficacy on health information anxiety among older adults

The findings support Hypothesis 3, confirming that information processing self-efficacy effectively alleviates health information anxiety among older adults. The findings revealed that information processing self-efficacy demonstrates superior efficacy in alleviating information-driven anxiety, technology-induced anxiety, and environment-triggered anxiety compared to self-perceived anxiety. According to the Conservation of Resources theory, individuals with abundant resources are less vulnerable to resource loss and are better able to acquire additional resources; in contrast, those with limited resources face a higher risk of resource depletion (114). Information processing self-efficacy, as a key psychological resource, helps individuals develop positive self-perceptions of their capabilities, reduce the depletion associated with negative emotions such as anxiety and frustration, and thereby promote problem-solving behaviors and the enhancement of positive emotions, facilitating the formation of a “resource gain spiral (115).”

“I used to get headaches when seeing medical terms I didn't understand. Now I search for some popular science videos to watch, and it feels both easy and fun.” (Interview G) “Sometimes when a webpage wouldn't load, I'd get really anxious. Now I try reconnecting or restarting the router, and I don't panic as much anymore.” (Interview H) “As I've gotten older, I wasn't comfortable posting online, afraid I might say something wrong. But then my child encouraged me to share about my daily fitness routines. To my surprise, my little tips were quite well-received, and I enjoy chatting with everyone every day.” (Interview I)

Older adults with higher information processing self-efficacy perceive greater control over health information, which reduces feelings of powerlessness and anxiety when faced with complex information and promotes a sense of social integration and belonging. These have the potential to enhance the mental health of older adults (116).

### 5.4 The mediating effect of information processing self-efficacy in the relationship between digital feedback and health information anxiety among older adults

The findings support Hypothesis 4, confirming that increased digital feedback significantly enhances information processing self-efficacy among older adults, consequently reducing their health information anxiety. As social participation naturally declines with age, the family emerges as the primary living sphere for older adults (47). Digital feedback, as a sustainable family-based social support resource, addresses the tripartite challenges of digital technology among older adults—device inaccessibility, skill deficiency, and technological apprehension (32)—and creates critical opportunities for older adults to engage with online health information. The intervention not only facilitates the accumulation of digital information skills and practical experience (77), enabling their transformation from information “passive recipients” to “active explorers” and from “information-disadvantaged” to “information-empowered” individuals (117), but also fundamentally reshapes their digital cognition, thought processes, and behavioral patterns (87). Concurrently, digital feedback fosters emotional fulfillment and cultivates positive affective states (118). Studies have shown that emotional support stimulates prefrontal cortex activation while attenuating negative emotional responses (119), thereby making older adults more likely to approach health information with a positive attitude and effectively strengthening their information processing self-efficacy. This enhanced self-efficacy contributes to cognitive reserve accumulation and amplifies motivation for novel knowledge acquisition, establishing a self-reinforcing cycle (120). Older adults with heightened information processing self-efficacy demonstrate greater confidence in confronting challenges and exhibit an increased propensity to leverage multimodal media resources for health information identification, integration, and application to refine health management strategies. Conversely, those with diminished self-efficacy frequently experience reduced self-assessment capacity and compromised self-worth perceptions, culminating in anxiety manifestations (56). Therefore, improving older adults' information processing self-efficacy through digital feedback represents a critical pathway to enabling their access to digital dividends and improving both physical and mental health outcomes. To achieve this goal, policy-making and social service practices can focus on promoting the development of a digitally supportive network that integrates family and community efforts. For instance, family-involved digital skills training programs can be incorporated into community-based older adults care services, fully leveraging the foundational role of the family in digital feedback.

### 5.5 Differential effects of the mediation model

Given that this study was conducted in China, the applicability of the mediation model may vary across different countries due to differences in sociocultural contexts and technological support environments. On the sociocultural level, Chinese society is characterized by a strong tradition of “family culture,” emphasizing familial bonds and collectivist values, as well as harmonious relationships among family members (121). As a result, older adults generally exhibit high trust in information provided by their children, positioning the family as the central context for digital feedback. Children's active assistance in helping parents acquire digital skills is not only perceived as technical support but also as an expression of filial piety and emotional bonding, which further strengthens the positive impact of digital feedback on alleviating older adults' health information anxiety. In contrast, in some Western societies that emphasize individualism, older adults tend to seek help from peers or professional institutions. For example, research by the University of the Third Age (U3A) Network Victoria shows that information and communication technology (ICT) courses are among the most popular programs, and because peer groups share similar life experiences in ICT use, older adults feel more at ease and relaxed during the learning process (122). On a technical level, China has established one of the world's leading and largest information and communication networks, providing robust technical support for the widespread implementation of digital feedback. However, in countries or regions with limited technological resources, issues such as unstable internet access and low digital device penetration severely restrict the frequency and continuity of digital feedback (123). Therefore, the mediation model proposed in this study is particularly relevant in societies with strong family cultures and rapid digital advancement. However, its effectiveness may be significantly diminished in regions where family support is weak or where the technological environment poses severe constraints.

Beyond the macro-level influences of cultural and technological environments on the mediation model, individual differences among older adults are equally non-negligible. Although the study assumes a certain level of homogeneity in older adults' digital experiences, in reality, there may be significant heterogeneity in their actual digital experiences. Regression results indicate that age and education level serve significant roles in the mechanism by which digital feedback influences health information anxiety among older adults, a finding consistent with prior research (31). Gerontological studies demonstrate that older adults experience progressive deterioration in basic cognitive functions such as perception and memory with advancing age, which significantly diminishes their capacity to accept and operate new things. This effect is particularly pronounced among older adults aged 80 and above, who typically require more granular demonstrations, extended repetitive learning periods, and sustained practice when receiving digital instruction from their children. Frequent memory lapses and operational errors often exacerbate self-doubt and anxiety in this population. In contrast, older adults under the age of 80 demonstrate superior cognitive preservation, enabling more proficient mastery of digital skills and consequently exhibiting stronger self-efficacy with correspondingly lower anxiety levels (124). Concurrently, older adults with lower education levels may experience difficulties in receiving digital instruction from their children due to deficits in foundational knowledge and technical comprehension. These challenges often engender frustration or resistance, thereby impeding their comprehension and application of health information (125). In contrast, older adults with higher education levels demonstrate superior learning capacity and a robust knowledge base, enabling more facile acquisition of novel skills and accelerated adaptation to technological environments. Moreover, some studies have also indicated that the impact of digital feedback on health information anxiety may vary among older adults with different health conditions. Older adults with poorer health, often experiencing illness or physical discomfort, tend to endure greater psychological stress, which may impair their concentration and reduce learning efficiency when receiving digital feedback (30). At the same time, they are more sensitive to health-related information and prone to associate its content with their own conditions, potentially exacerbating their health information anxiety (126). In contrast, older adults with better health generally have more favorable physical and mental states, stronger motivation to learn, and are more likely to acquire digital skills effectively with the support of their children, enabling them to process health information more rationally.

## 6 Conclusions

The theoretical contribution of this study lies in the following aspects: first, this study integrates digital feedback, information processing self-efficacy, and health information anxiety into a unified research framework, enriching the application of social support theory, self-efficacy theory, and information ecology theory in the digital era. It provides theoretical foundations and policy entry points for promoting the coordinated development of digital inclusion and mental health among older adults. Second, by examining digital feedback across its three dimensions, the study validates the functional extension of digital feedback within health information contexts, offering an innovative approach to alleviating older adults' health information anxiety from a familial perspective. Third, it reveals the mediating role of information processing self-efficacy in the relationship between digital feedback and health information anxiety, providing a novel perspective on how intergenerational support influences older adults' mental health, thereby advancing both scholarly comprehension and practical implementation regarding this issue.

Furthermore, the following practical and policy implications have been identified: first, community and university resources should be fully mobilized to address older adults' most frequent health information needs through regular digital teaching activities or lectures, actively encouraging children or other younger family members to participate together with older adults, to foster a family-friendly digital learning environment. Second, children should remain patient during the process of digital feedback, demonstrating understanding and tolerance for older adults' habits and learning pace. This involves breaking down complex technological tasks into simple, manageable steps and progressing gradually from basic to advanced functions. At the same time, they should encourage older adults to ask questions and express their thoughts, enabling tailored support based on actual needs and promoting mutual understanding and reciprocal learning between generations. Third, digital feedback should extend beyond mere access to technology to include skill transfer and digital literacy development, ensuring its effectiveness and sustainability. While teaching digital skills, efforts should also focus on cultivating older adults' critical evaluation of health information. Ultimately, older adults should adopt a proactive approach to digital engagement, openly discussing their challenges with digital technologies with their children and reinforcing newly acquired skills through consistent practice in everyday life.

The study also has several limitations: first, it relies on questionnaire surveys with a single data source, which may be subject to social desirability bias or recall bias, potentially affecting the accuracy and objectivity of the data. Second, the cross-sectional design prevents the examination of longitudinal developmental patterns in the relationships among digital feedback, information processing self-efficacy, and health information anxiety, thereby limiting causal inferences. Third, this study only considers “information processing self-efficacy” as a mediating variable; the potential roles of other mediators—such as digital literacy or emotional support—remain to be explored. Fourth, the qualitative sample size is relatively small, limiting the representativeness of the sample. Fifth, heterogeneity analyses based on control variables such as age group, health status, number of children, and living arrangements have not been conducted, which may obscure differences in digital experiences within the older adult population. Sixth, the study's findings are derived from a specific cultural and technological context, and their generalizability may be constrained in settings with different cultural norms or levels of technological development.

Future research directions: first, to enhance the robustness of findings, future studies should employ longitudinal tracking of samples and incorporate observational or behavioral data for triangulation, enabling an investigation of the dynamic interplay among digital feedback, information processing self-efficacy, and health information anxiety. Second, research should expand in scope by increasing sample sizes and integrating systematic in-depth interviews to thoroughly explore the impact of older adult population heterogeneity on the proposed model, while also testing multiple mediating pathways—such as digital literacy and emotional support—to improve the representativeness and generalizability of the findings.

## Data Availability

The raw data supporting the conclusions of this article will be made available by the authors, without undue reservation.

## Supplementary material

Supplementary file S1 Questionnaire.

Supplementary file S2 Interview outline.
