# Supplementary material for: The mechanism of digital feedback on health information anxiety among older adults: information processing self-efficacy as a mediating variable
Source: Front Public Health. 2025 Nov 5;13:1676970. doi: 10.3389/fpubh.2025.1676970 (PMC12626806; doi:10.3389/fpubh.2025.1676970)
Supplement: Supplementary file S1 — Questionnaire. [file Table_1.docx]

**Survey on the Mechanism of Digital Feedback on Health Information Anxiety among the Elderly**

**Dear sir/madam:**

Hello! Thank you for sparing your valuable time to complete this questionnaire! This survey is academic and aims to examine the mechanism by which digital feedback affects health information anxiety in the elderly. The questionnaire is anonymous, and all data will be used exclusively for this research paper. We sincerely thank you for your support and cooperation!

According to the health maintenance needs of the elderly, the health information referred to in this questionnaire mainly includes four types: medical services (e.g., disease consultation, telemedicine), rehabilitation (e.g., medication, rehabilitation precautions), disease prevention (e.g., disease etiology, preventive measures, early symptoms), and health preservation (e.g., dietary hygiene, nutrition).

There are no right or wrong answers to this questionnaire. Please select the appropriate options based on your actual situation. This may take you approximately 3-5 minutes. We will strictly keep your response information confidential. Your cooperation and support will contribute to academic research. Thank you again for your help!

**Part 1: Basic Personal Information**

1. **Your gender**

○Male ○Female

1. **Your age**

○ 60-69 years ○ 70-79 years ○ 80 years and above

1. **Your education level**

○ Primary or below ○ Junior high ○ Specialized Secondary/Senior high ○ Junior college or above

1. **Your economic status**

○ Very poor ○ Poor ○ Average ○ Good ○ Excellent

1. **Your residence area**

○Rural ○Urban

1. **Number of your children**

○ Childless or no living children (skip to the end of the questionnaire and submit the answer)

○ 1 ○ 2 ○ 3 ○ 4 and above

1. **Your Living arrangement**

○ Living alone ○ With spouse only ○ With children only ○ With spouse and children ○ With other relatives

1. **Your health status**

○ Very poor ○ Poor ○ Average ○ Good ○ Excellent

**Part 2: Scale items**

The following questionnaire uses the Likert scale method, where "1" to "7" represent "very non-conforming" to "very conforming". Please answer according to your actual situation. There are no right or wrong answers, so please respond without concern.

1. **Digital Feedback Questionnaire**

|  | very non-conforming | non-conforming | somewhat non-conforming | neutral | somewhat conforming | conforming | very conforming |
| --- | --- | --- | --- | --- | --- | --- | --- |
| Your children help you establish internet connectivity. | ○ | ○ | ○ | ○ | ○ | ○ | ○ |
| Your children help you acquire digital devices (e.g., smartphones). | ○ | ○ | ○ | ○ | ○ | ○ | ○ |
| Your children help you download and install applications. | ○ | ○ | ○ | ○ | ○ | ○ | ○ |
| Your children teach you to master searching and obtaining information skills through multiple channels(e.g., search engines, WeChat official accounts). | ○ | ○ | ○ | ○ | ○ | ○ | ○ |
| Your children teach you to master evaluating and selecting high-quality content skills. | ○ | ○ | ○ | ○ | ○ | ○ | ○ |
| Your children teach you to master information application skills for problem-solving. | ○ | ○ | ○ | ○ | ○ | ○ | ○ |
| Your children teach you to master digital information transmission, sharing, and social interaction skills through digital platforms (e.g., WeChat, Weibo, Douyin). | ○ | ○ | ○ | ○ | ○ | ○ | ○ |
| Your children encourage your participation in digital environments and enhance your willingness to exposure and adopt technologies. | ○ | ○ | ○ | ○ | ○ | ○ | ○ |
| Your children explain the significance, life impacts, and future development of digital technologies to deepen your awareness and understanding. | ○ | ○ | ○ | ○ | ○ | ○ | ○ |
| Your children share your knowledge about cybersecurity risks (e.g., online fraud, privacy protection). | ○ | ○ | ○ | ○ | ○ | ○ | ○ |
| Your children guide you in adopting appropriate digital ethics principles and behavioral norms (e.g., respecting privacy, safeguarding intellectual property rights). | ○ | ○ | ○ | ○ | ○ | ○ | ○ |

1. **Information Processing Self-Efficacy Questionnaire**

|  | very non-conforming | non-conforming | somewhat non-conforming | neutral | somewhat conforming | conforming | very conforming |
| --- | --- | --- | --- | --- | --- | --- | --- |
| You habitually use the internet as a primary information acquisition channel. | ○ | ○ | ○ | ○ | ○ | ○ | ○ |
| You can quickly familiarize yourself with various information platforms (e.g., websites, official accounts, application software). | ○ | ○ | ○ | ○ | ○ | ○ | ○ |
| You adapt to using different search methods (e.g., keyword search, voice search, image recognition search) to obtain information. | ○ | ○ | ○ | ○ | ○ | ○ | ○ |
| You have exerted substantial effort to acquire useful information. | ○ | ○ | ○ | ○ | ○ | ○ | ○ |
| You have invested considerable effort to resolve difficulties encountered during information processing. | ○ | ○ | ○ | ○ | ○ | ○ | ○ |
| You have devoted significant effort to improving information processing efficiency. | ○ | ○ | ○ | ○ | ○ | ○ | ○ |
| You can effortlessly access needed information. | ○ | ○ | ○ | ○ | ○ | ○ | ○ |
| You can make correct decisions based on obtained information. | ○ | ○ | ○ | ○ | ○ | ○ | ○ |
| When encountering information processing difficulties, you can generate multiple solutions. | ○ | ○ | ○ | ○ | ○ | ○ | ○ |
| You possess more effective information than others. | ○ | ○ | ○ | ○ | ○ | ○ | ○ |
| The information you share benefits others. | ○ | ○ | ○ | ○ | ○ | ○ | ○ |

1. **Please choose option B for this question**

○ A ○ B ○ C ○ D

1. **Health Information Anxiety Questionnaire**

|  | very non-conforming | non-conforming | somewhat non-conforming | neutral | somewhat conforming | conforming | very conforming |
| --- | --- | --- | --- | --- | --- | --- | --- |
| You often misinterpret or exaggerate health information, feeling that your own or your family's health is at risk. | ○ | ○ | ○ | ○ | ○ | ○ | ○ |
| You often browse health information uncontrollably and constantly refresh to obtain satisfactory information. | ○ | ○ | ○ | ○ | ○ | ○ | ○ |
| You often feel anxious about missing health information that others may have received. | ○ | ○ | ○ | ○ | ○ | ○ | ○ |
| You often spend too much time on health information and feel guilty and distressed as a result. | ○ | ○ | ○ | ○ | ○ | ○ | ○ |
| You worry about whether the health information you obtain is authentic. | ○ | ○ | ○ | ○ | ○ | ○ | ○ |
| You are concerned that the health information you access is unclear or difficult to understand. | ○ | ○ | ○ | ○ | ○ | ○ | ○ |
| You worry about contradictions or conflicts in health information from different sources. | ○ | ○ | ○ | ○ | ○ | ○ | ○ |
| You are concerned that the amount of health information exceeds your ability to receive and process it. | ○ | ○ | ○ | ○ | ○ | ○ | ○ |
| You worry about being misled by homogeneous health information pushed by platforms. | ○ | ○ | ○ | ○ | ○ | ○ | ○ |
| When accessing health information platforms, you feel disappointed and frustrated if information fails to load, links break, or network errors occur. | ○ | ○ | ○ | ○ | ○ | ○ | ○ |
| When health information platforms are incompatible, you feel at a loss. | ○ | ○ | ○ | ○ | ○ | ○ | ○ |
| When platform interfaces (e.g., fonts, colors, layouts) are overly complex or disordered and incompatible with your visual and operational habits, you feel annoyed and impatient. | ○ | ○ | ○ | ○ | ○ | ○ | ○ |
| When health information platforms fail to meet your needs in terms of functions and services, you feel worried. | ○ | ○ | ○ | ○ | ○ | ○ | ○ |
| You often worry about ineffective online regulation leading to personal information leakage. | ○ | ○ | ○ | ○ | ○ | ○ | ○ |
| You often worry about facing discrimination or exclusion when sharing health information or expressing health-related opinions. | ○ | ○ | ○ | ○ | ○ | ○ | ○ |
| You often worry that health information may be exaggerated or distorted during dissemination, causing public opinion to deviate from facts. | ○ | ○ | ○ | ○ | ○ | ○ | ○ |
