# Supplementary material for: The mechanism of digital feedback on health information anxiety among older adults: information processing self-efficacy as a mediating variable
Source: Front Public Health. 2025 Nov 5;13:1676970. doi: 10.3389/fpubh.2025.1676970 (PMC12626806; doi:10.3389/fpubh.2025.1676970)
Supplement: Supplementary file S2 — Interview outline. [file Table_2.docx]

**Interview Outline on the Mechanism of Digital Feedback on Health Information Anxiety among the Elderly**

**Dear sir/madam:**

Hello! I am XX from XX College of XX University, and I am currently conducting a study on the mechanism of digital feedback on health information anxiety among the elderly. This study seeks to understand your views on the development of digital technologies, as well as your experiences and evaluations when receiving digital feedback from your children and processing health information through the internet. Your real experiences and thoughts are crucial to our study!

The interview will take approximately 30 minutes. There are no right or wrong answers, and you are encouraged to express your thoughts freely. You may skip any questions you do not wish to answer. With your consent, the conversation will be recorded to ensure accurate documentation of the discussion. All information provided will be kept strictly confidential and used solely for academic research purposes. Personal details such as your name and address will not be disclosed in the reports. Participation is entirely voluntary, and you may terminate the interview at any time.

Thank you very much for your participation and cooperation! May we ask if you voluntarily agree to participate in this in-depth interview survey? (If yes, a consent form will be provided and signed to formally begin the in-depth interview.)

**1. What are the basic personal details of the survey respondents (age, education level, number of children, residence area, living arrangement, economic status, health status)?**

**2. Why did you choose to use the internet to process health information?**

**3. What difficulties have you encountered while using the internet to process health information? How did you resolve them? Have you experienced anxiety in this process?**

**4. While using the internet to process health information, has your child provided you with assistance? If yes, could you provide a few examples? (If no, please skip directly to question 8.)**

**5. How do you feel when receiving such help from your children?**

**6. Through this kind of assistance from your children, do you feel more confident about your ability to process online health information? In what ways is this confidence manifested?**

**7. Do you think that the anxiety you experience while processing online health information has been alleviated through this kind of assistance from your children?**

**8. While using the internet to process health information, in what other aspects would you like to receive assistance from your children?**

**9. Is there any additional content you wish to add regarding this interview?**
